# Isx9 Regulates Calbindin D28K Expression in Pancreatic β Cells and Promotes β Cell Survival and Function

**DOI:** 10.3390/ijms19092542

**Published:** 2018-08-27

**Authors:** Julien B. Pujol, Eija Heikkila, Claudia Savoia, Asghar Hajibeigi, Umberto De Marchi, Pavan K. Battiprolu, Orhan K. Öz, El Hadji M. Dioum

**Affiliations:** 1 Department of Cell Biology, Nestle Institute of Health Sciences, EPFL Campus, 1015 Lausanne, Switzerland; julienpujol@hotmail.com (J.B.P.); eijaheikkila7@gmail.com (E.H.); claudia.savoia@rd.nestle.com (C.S.); Umberto.DeMarchi@rd.nestle.com (U.D.M.); 2 Department of Radiology, University of Texas Southwestern Medical Center, Dallas, TX 75390, USA; Asghar.Hajibeigi@UTSouthwestern.edu (A.H.); Pavan.battiprolu@yahoo.com (P.K.B.); Orhan.Oz@UTSouthwestern.edu (O.K.Ö.)

**Keywords:** Isx9, apoptosis, calbindin-D28K, calcium homeostasis, inflammation, serum deprivation, calcineurin, NFAT transcription factor, β cell function

## Abstract

Pancreatic β-cell dysfunction and death contribute to the onset of diabetes, and novel strategies of β-cell function and survival under diabetogenic conditions need to be explored. We previously demonstrated that Isx9, a small molecule based on the isoxazole scaffold, drives neuroendocrine phenotypes by increasing the expression of genes required for β-cell function and improves glycemia in a model of β cell regeneration. We further investigated the role of Isx9 in β-cell survival. We find that Isx9 drives the expression of Calbindin-D28K (D28K), a key regulator of calcium homeostasis, and plays a cytoprotective role through its calcium buffering capacity in β cells. Isx9 increased the activity of the calcineurin (CN)/cytoplasmic nuclear factor of the activated T-cells (NFAT) transcription factor, a key regulator of D28K, and improved the recruitment of NFATc1, cAMP response element-binding protein (CREB), and p300 to the D28K promoter. We found that nutrient stimulation increased D28K plasma membrane enrichment and modulated calcium channel activity in order to regulate glucose-induced insulin secretion. Isx9-mediated expression of D28K protected β cells against chronic stress induced by serum withdrawal or chronic inflammation by reducing caspase 3 activity. Consequently, Isx9 improved human islet function after transplantation in NOD-SCID mice in a streptozotocin-induced diabetes model. In summary, Isx9 significantly regulates expression of genes relevant to β cell survival and function, and may be an attractive therapy to treat diabetes and improve islet function post-transplantation.

## 1. Introduction

Diabetes mellitus is a growing public health issue, characterized by dysfunctional and/or loss of insulin-producing β cells; hence the urgent need for therapeutic agents that can promote function and maintenance of β cells. Type 1 diabetes is characterized by loss of pancreatic β cells due to autoimmunity and inflammation [1], while increased peripheral insulin resistance and β cell dysfunction characterized type 2 diabetes [2]. Under physiological conditions, postprandial glucose metabolism in the β cells leads to elevated intracellular [ATP]/[ADP] ratio, closure of KATP channels, and membrane depolarization, leading to activation of the voltage-dependent Ca^2+^ channels causing a rise in intracellular calcium concentrations ([Ca^2+^]_i_), which in turn triggers insulin release [3,4]. In this context, calcineurin (CN), or serine/threonine phosphatase (PP2B), is activated by Ca^2+^/calmodulin [5]. Activation of CN is required for the dephosphorylation and NFAT family of transcription factors NFATc1, c2, c3 and c4 [6]. Therefore, the CN/NFAT signaling couples intracellular calcium rise to transcription of genes involved in β cell function and survival [7]. Conditional deletion of the CN regulatory subunit (Cnb1) in β cells leads to reduced β cell mass and function, with the ultimate development of diabetes in the mice [8]. Furthermore, patients treated with immunosuppressant inhibitor of CN such as cyclosporine and tacrolimus (FK506) have a higher prevalence of β cell failure and diabetes. Taken together, these data suggest that CN/NFAT signaling regulates genes required for β cell function and maintenance [8,9].

We recently identified Calbindin D28K (D28K), an EF-hand Ca^2+^-binding protein, as an NFAT target gene in β cells [10]. D28K is a cytosolic protein with a strong calcium buffering capacity that prevents the cytotoxic effect of a high concentration of free calcium in several tissues [11,12]. In pancreatic β cells, D28K blunts insulin secretion induced by KCl depolarization [13], in part through its buffering capacity and possibly by inhibition of the L-type voltage gated calcium channel activity [14]. Additionally, D28K has anti-apoptotic properties in cells treated with pro-inflammatory cytokines [13,14]. Numerous studies have shown that D28K, by its Ca^2+^ buffering capacity, regulates [Ca^2+^]_i_ in response to physiological stimuli and protects against calcium-mediated cellular toxicity [15,16,17]. Recent studies have emphasized the anti-apoptotic properties of D28K via reduction of caspase 9 and caspase 3 activity in both neurons and β cells [15,18,19,20].

Apoptosis is probably the main form of β cell death in patients with type 1 diabetes [21]. Cytokine signaling is regulated by intracellular signals like nitric oxide production and disruption of mitochondrial function, leading to cytochrome c release, which recruits and activates caspase 9 and caspase 3/7, which induces apoptosis in both type 1 and type 2 diabetes [22]. Growth factor signaling, in particular through the insulin and/or IGF1 receptors signaling, regulates β cell function, survival, and proliferation [23,24,25], and the PI3-kinase/AKT signaling plays a critical role in this process [26]. Serum deprivation or serum free medium (SFM) treatment can be used to induce oxidative stress and impairment of mitochondrial function.

The small molecule *N*-cyclopropyl-5-(thiophen-2-yl)-isoxazole-3-carboxamide (Isx9), previously identified in a high throughput screen as NKX2.5 activator [27], was described as a positive regulator of transcription factors involved in β cell differentiation and function. Isx9 increased p300/CBP histone acetyl transferase (HAT) activity, leading to hyperacetylation of histones H3/H4, and increased the expression of islet-specific factors coordinating β cell differentiation and function in human and mouse islets [28,29]. Treatment of Isx9 increased insulin gene expression in human islet cells, improved insulin secretion [28], and protected glucose-responsive signaling pathways under lipotoxic conditions [29]. Amongst the genes upregulated by Isx9, NeuroD1, insulin, and glucokinase are in part regulated by CN/NFAT signaling [7,8]. In neurons, Isx9 induces intracellular Ca^2+^ rise via the voltage-gated calcium channel and the NMDA receptor [27]. Considering that NFAT upregulated D28K expression [10], we investigated the role of Isx9 in Ca^2+^ homeostasis and in the regulation of D28K expression in β cells. We further investigated the cytoprotective role of Isx9 against chronic serum withdrawal or inflammation. The high probability of β cell loss observed after islet transplantation in diabetic patients [30] calls for efficacious strategies to improve their function and survival. Here, we showed that Isx9 presented a therapeutic method to improve β cell function and survival.

## 2. Results

### 2.1. Isx9 Increases Expression Levels of NFAT and D28K

**Figure 1:**
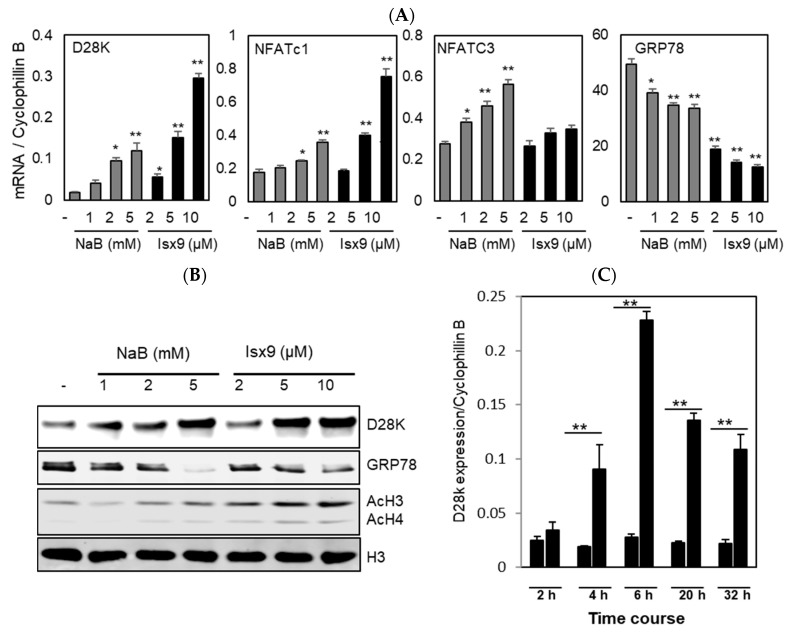

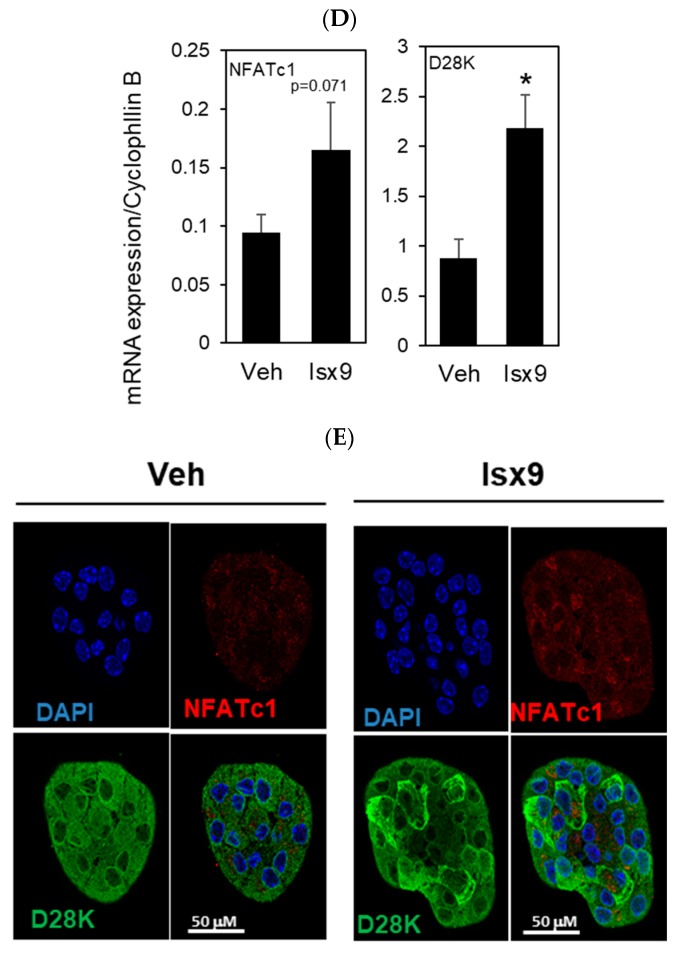
Sodium Butyrate and Isx9 activates calbindin D28K and nuclear factor of the activated T-cells (NFAT) expression in β-cells. (**A**) Dose response activation of Calbindin D28K, NFATc1, NFATc3, and GRP78 in MIN6 cells after 48 h treatment with 1, 2 and 5 mM sodium butyrate (NaB) or 2, 5 and 10 µM Isx9, * *p* < 0.05, ** *p* < 0.01 treatment relative to vehicle. (**B**) Immunoblot of D28K, GRP78, acetyl histones H3 K9/K14 and H4 (K5/8/12/16) with total histone H3 used as a loading control from whole cell lysate of MIN6 cells treated with increasing doses of NaB and Isx9 for 48 h. (**C**) Time course of Isx9 (10 μM) induced activation of the Calbindin D28K gene expression in INS1E cells cultured in complete medium (10% FBS). Data presents as Mean ± SEM of three independent experiments ** *p* < 0.01 relative to control cells. (**D**) Expression of D28K and NFATc1 measured by qPCR and in mouse primary islets after 24 h treatment with 10 µM Isx9. Data presented as mean + SEM of three independent experiments * *p* < 0.05. (**E**) Immunohistochemical staining of nuclei (DAPI), NFATc1, and D28K in primary mouse islets monolayer cultures after 10 µM Isx9 treatment for 48 h (Scale bar, 50 μm).

NanoString nCounter technology [31] was used to measure the expression of selected genes in human islets treated with Isx9 (Figure S1A). Isx9 increased the expression of D28K, NFATc1, and NFATc2, but also genes involved in calcium homeostasis such as the L-type calcium channels Cav2.3 and K_ATP_ channel subunits (KCNB1 and ABCC8). Isx9 also increased the expression of several genes involved in β cell function and survival (IER3, D28K, IGFBP5, CXCL12, CXCR4 CCNDA, and CDKN2c) and reduced the expression of Grp78, a marker of cellular stress (Figure S1A). Considering the role of NFAT in D28K expression [10], these data suggested that Isx9 could participate in calcium homeostasis and NFAT/D28K signaling in β cells. To further investigate the role of Isx9 in D28K expression and signaling, we used rodent pancreatic β cell lines MIN6 and INS1E as cellular models. Based on previous reports [32], we used sodium butyrate (NaB) as a positive control for increased D28K expression in MIN6 cells. Comparable to NaB, Isx9 upregulated the expression of D28K and NFATc1 in a dose dependent manner from 0 to 10 µM in MIN6 cells and reduced the protein expression of the ER stress marker GRP78 (Figure 1A). Furthermore, Isx9 significantly increased NFATc1, NFATc2, NFATc3 and NFATc4, but not NFAT5 expression in INS1E cells (Figure S1B). The upregulation of D28K mRNA and protein expression showed the same trend in a dose-dependent manner after NaB or Isx9 treatment, which was accompanied by an increase in Histone H3 and H4 acetylation (Figure 1B). At the transcriptional level, Isx9 significantly increased D28K transcription as early as 4 h (Figure 1C). In primary mouse islet, Isx9 increased D28K and NFATc1 expression in at the mRNA (Figure 1D). Furthermore, immunohistochemical staining of mouse islets after Isx9 treatment showed an increase in NFATc1 and D28K protein expression; however, D28K is not uniform in all cells (Figure 1E).

### 2.2. Isx9 Increases NFAT Transcriptional Activity and Recruitment of the Transcriptional Complex

**Figure 2:**
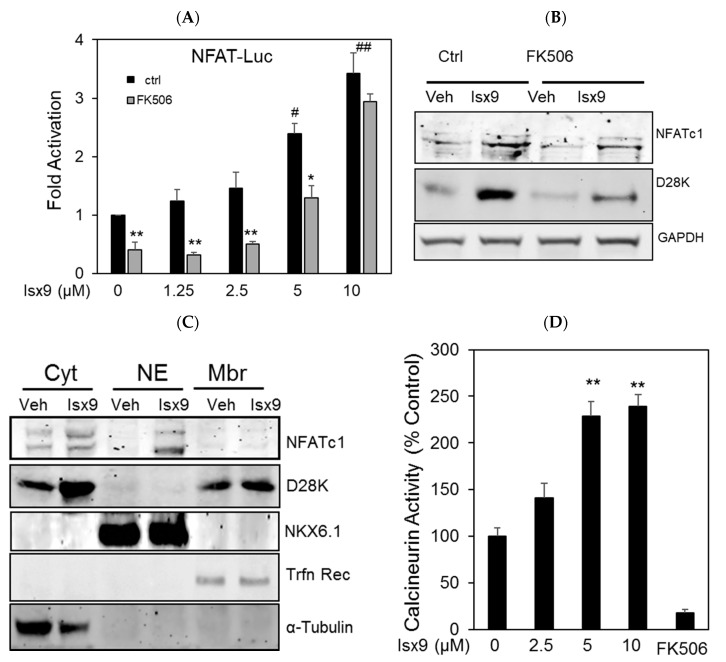

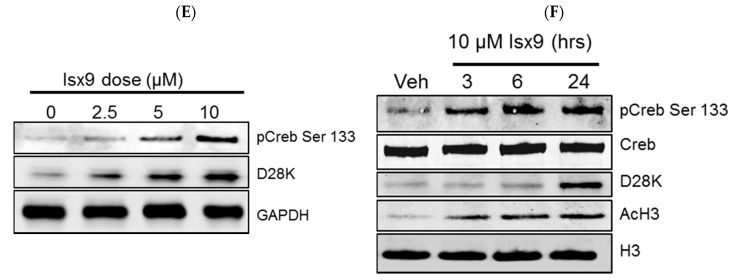
Isx9 increased NFAT transcriptional activity. (**A**) Dose-dependent activation of the NFAT reporter in β cells after 24 h treatment with increasing dose of Isx9 in the presence or absence of 0.3 µM FK506, mean ± SEM of three independent experiments in triplicates, # *p* < 0.05 and ## *p* < 0.01 Isx9 versus non-treated cells; * *p* < 0.05, ** *p* < 0.01 effect of FK506 treatment versus control for each Isx9 dose. (**B**) Immunoblotting of NFATc1 and D28K in MIN6 whole cell extract after 48 h treatment with vehicle DMSO (Veh) or 10 µM Isx9 in the presence or absence of calcineurin inhibitor FK506. (**C**) Subcellular fractionation (Pierce) of MIN6 cells treated with Isx9 or vehicle into cytoplasmic (Cyt), nuclear (NE) and membrane (Mbr) fractions followed by immunoblotting of NFATc1 and D28K. α-Tubulin, Nkx6.1, and Transferrin receptors are used as loading controls. (**D**) Calcineurin activity in MIN6 represented as % of untreated cells treated with increasing doses of Isx9, FK506 is used as a negative control, mean ± SEM of three independent experiments in triplicates, ** *p* < 0.01 vs. control. (**E**) Immunoblotting of phospho-Creb1-Ser 133, D28K, and GAPDH after increasing dose of Isx9 for 24 h or (**F**) after 8 h and 24 h treatment with 10 µM Isx9 in MIN6 cells.

NFATc1 or NFATc2 ectopic overexpression was shown to upregulate D28K expression in MIN6 cells [10]. However, under physiological conditions, NFAT activity is post translationally regulated by calcineurin. To determine if induction of D28K expression is secondary to Isx9 stimulated increase of NFAT transcriptional activity, we used the NFAT *cis*-Reporter system in MIN6 cells. Isx9 increased NFAT transcriptional activity nearly four-fold in a dose-dependent manner. In addition, higher concentrations of Isx9 blunted the inhibitory effect of FK506 (Figure 2A). CN/NFAT inhibition by FK506 reduced the Isx9 upregulation of D28K protein, but not NFATc1 in MIN6 cells (Figure 2B). Isx9 increased endogenous NFATc1 protein level, as well as its nuclear translocation (Figure 2B,C), suggesting a direct effect of Isx9 on calcineurin activity. Using an in vitro assay, Isx9 significantly increased calcineurin activity in a dose-dependent manner (Figure 2D), which might result in part from the Isx9-induced Ca^2+^ influx via the L-type calcium channel (Figure S2). Calcineurin was previously reported to activate Creb, a transcription factor required for β cell function and survival [33]. In β cells, Isx9 increased Creb1 phosphorylation in a dose- (Figure 2E) and time-dependent manner (Figure 2F).

**Figure 3:**
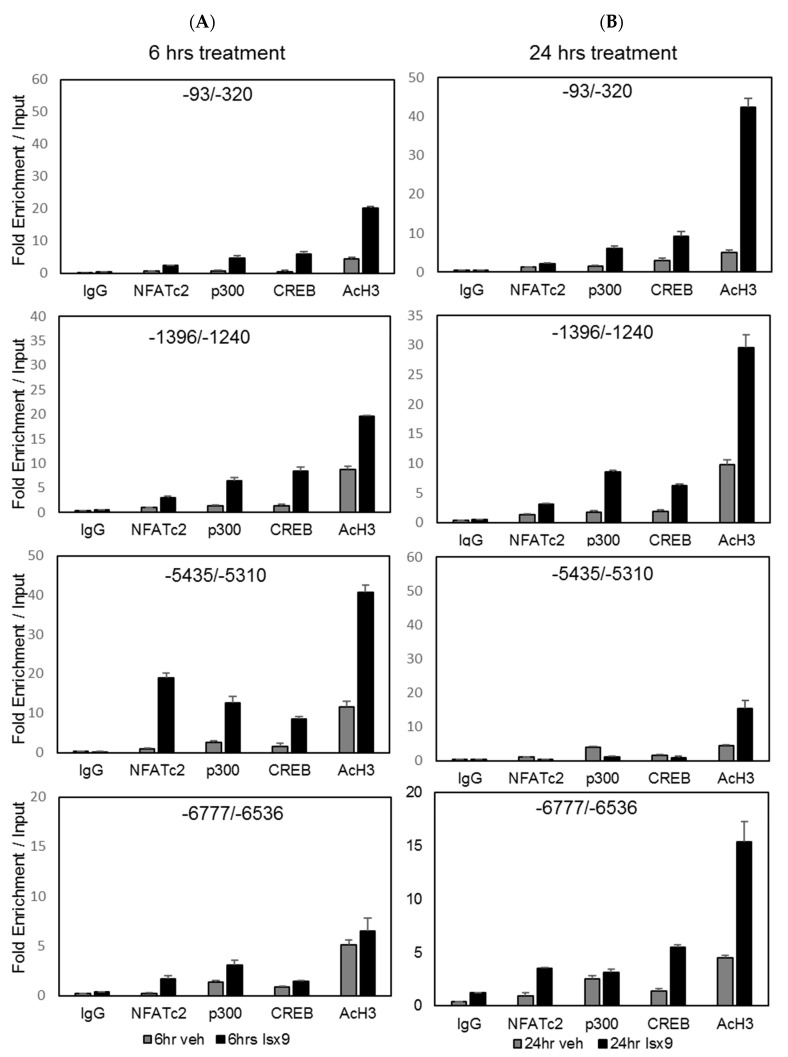
Isx9-increased transcription factors recruitment to the D28K promoter by ChIP-assay. (**A**) Chromatin enrichment of NFATc2, Creb1, p300, and acetylated histone H3 (AcH3 K9/14) to the rat D28K promoter in INS1 E cells treated with 10 µM Isx9 for 6 h or (**B**) for 24 h at various regions of the rat D28K promoter (−7000/+219). Data presented as Mean ± SD of a representative experiment in sextuplet.

Phosphorylation of Creb1 at Ser133 promotes recruitment of the transcriptional co-activators CBP/p300 [34], leading to interactions with transcription factors, which contributes to transcriptional activation of target genes synergy [35,36]. As the D28K promoter contains several conserved CREB binding elements adjacent to NFAT binding sites (Figure S3), we measured transcription complex recruitment to the D28K promoter by ChIP-assay and assessed Isx9 contribution. We used NFATc1 in MIN6 (Figure S4A) and NFATc2 in INS1E cells (Figure 3), which express higher levels of the respective proteins. Isx9 increased recruitment of NFATc2, Creb1, and p300 to the proximal and distal D28K promoter as early as 6 h after treatment (Figure 3A), prior to increase in histone H3 acetylation seen after 24 h treatment (Figure 3B). In the distal promoter (−5435/−5310), the early recruitment of Creb1, p300 and NFATc2 induced by Isx9 was subsequently reduced after 24 h treatment (Figure 3B). Similarly, Isx9 also increased recruitment of NFATc1 and p300 to the mouse D28K core promoter (−36/+139) (Figure S4A). As Isx9 was shown to increase insulin transcription in human islets [28], we similarly found increased recruitment of NFAT/p300/Creb on the rat insulin 2 promoter (Figure S4B).

In summary, Isx9 increased D28K expression via calcineurin/NFAT activation and by promoting recruitment of the transcriptional complex (NFAT, Creb1, p300) to its proximal and distal promoter, which might be facilitated by histone H3 acetylation.

### 2.3. Isx9 Protects β Cells against Apoptosis through Upregulation of D28K

**Figure 4:**
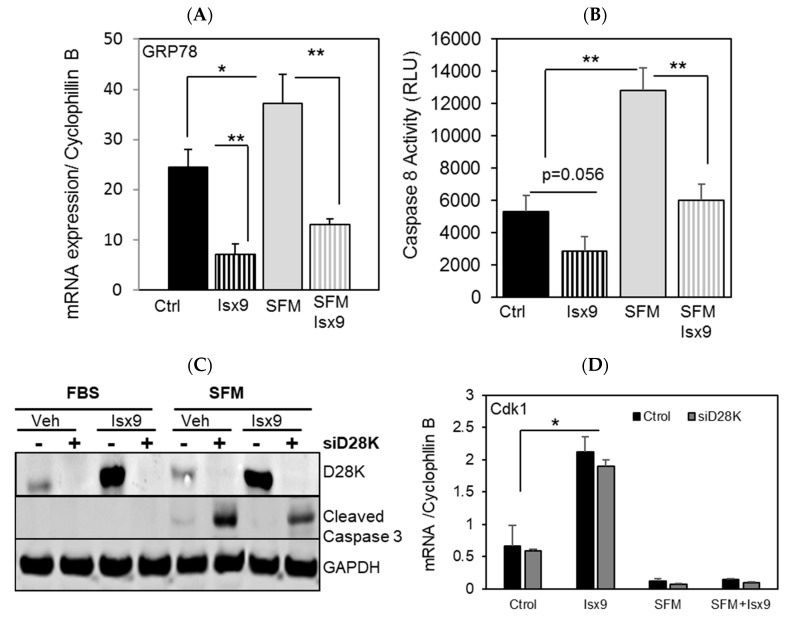

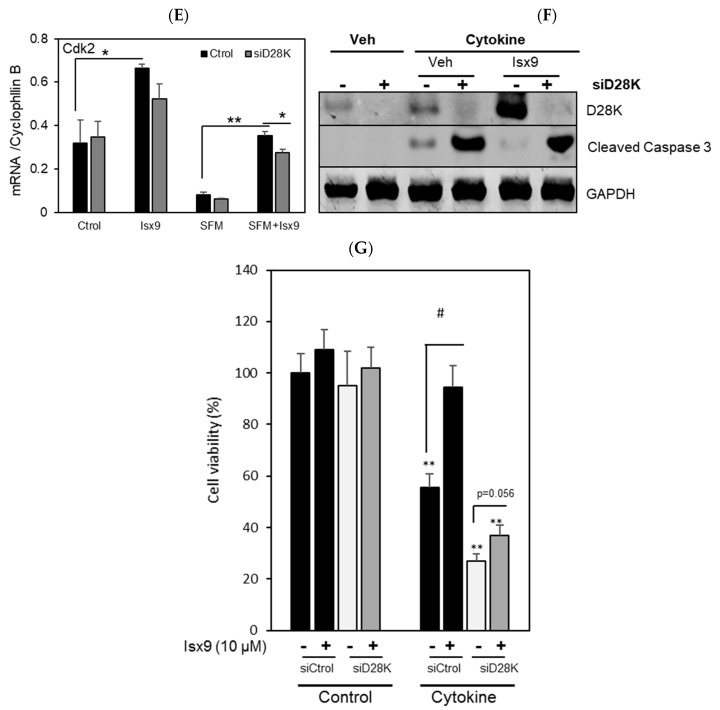
Isx9-protected β cells against apoptosis in part through D28K. qPCR analysis of (**A**) GRP78 expression and (**B**) caspase 8 activity in INS1 E cells treated with vehicle control (Ctrl) or Isx9 (10 μM) and serum free medium (SFM) treatment for 48 h. Data are presented as mean ± SEM from three independent experiments in triplicates, * *p* < 0.05 and ** *p* < 0.01. (**C**) Immunoblotting of D28K, cleaved caspase 3, and GAPDH or (**D**) expression of Cdkn1 a/p21 cip and (**E**) CDK2 by qPCR in INS1 E cells after siRNA knockdown of D28K (siD28K) and/or SFM treatment for 96 h in the presence or absence of Isx9.* *p* < 0.05 relative to control. (**F**) Immunoblotting of INS1 E cells treated with cytokine mix for 48 h in the presence or absence of Isx9 and upon D28K knockdown. (**G**) Effect of Isx9 on INS1 E cell viability represented as a percentage relative to control cells after treatment with a cytokine mix after D28K knockdown using Alamar Blue represented as mean + SEM of three independent experiments, * *p* < 0.05 and ** *p* < 0.01 relative to control vehicle treated cell, # *p* < 0.05.

Ischemia and reduced growth factor signaling together with inflammation represent stressors affecting pancreatic β cell function and survival in transplanted islets [37]. Considering the role of NFAT, Creb1 and D28K in cell survival, we investigated the role of Isx9 in β cell survival in response to stress. Serum withdrawal (SFM) was used as a model to induce apoptosis and dysfunction in β cell as it causes oxidative stress and impaired mitochondrial function [38,39]. In INS1 E cells, Isx9 significantly attenuated SFM-induced upregulation of GRP78 mRNA expression (Figure 4A) and caspase 8 activity (Figure 4B). To determine if the reduced stress response in Isx9 treated cells involved D28K, we depleted D28K protein by siRNA knockdown (Figure S5A). D28K knockdown increases apoptosis as assessed by cleaved caspase 3 levels in cells cultured in SFM (Figure 4C). SFM was shown to induce cell cycle arrest by affecting cyclin genes expression [40]. Isx9 increased the expression of cell cycle regulated genes such as cyclin A2 (Ccna2), D1 (Ccnd1), and E2 (Ccne1) (Figure S5C) and cyclin dependent kinases Cdk1 and Cdk2 (Figure 4D,E), which were reduced by SFM. Unlike Cdk1, Cdk2, a major regulator of β cell mass, was rescued by Isx9 following SFM treatment, in part through D28K expression (Figure 4E). It is worth noting that several Isx9-regulated genes, such as Chromogranins (ChgA, ChgB) (Figure S5B,C), Cdk1, and Cdk2 (Figure 4D,E), are known NFAT target genes [41]. Chronic inflammation, which occurs in type 1 diabetes, is known to increase caspase 3 activity and apoptosis in β cells. Isx9 reduces cleaved caspase 3 induced by inflammatory cytokines (IL1β, TNFα, IFNγ) through D28K overexpression induced by Isx9 (Figure 4F). Chronic treatment with pro-inflammatory cytokine mix reduced β cell viability, which was exacerbated by D28K siRNA knockdown (Figure 4G). Isx9 significantly improved β cell survival in response to chronic inflammation significantly through D28K. While it is likely that other factors activated by Isx9 contribute to the cytoprotective properties of Isx9, these results suggest that the cytoprotective properties of Isx9 in β cells were substantially mediated by increased D28K expression.

### 2.4. Isx9 Protects against SFM-Induced β Cell Dysfunction

**Figure 5:**
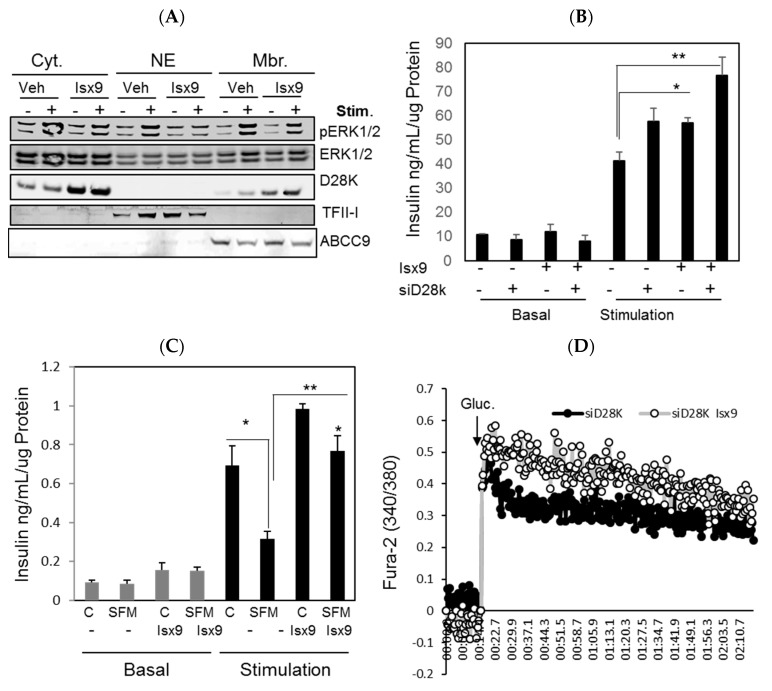

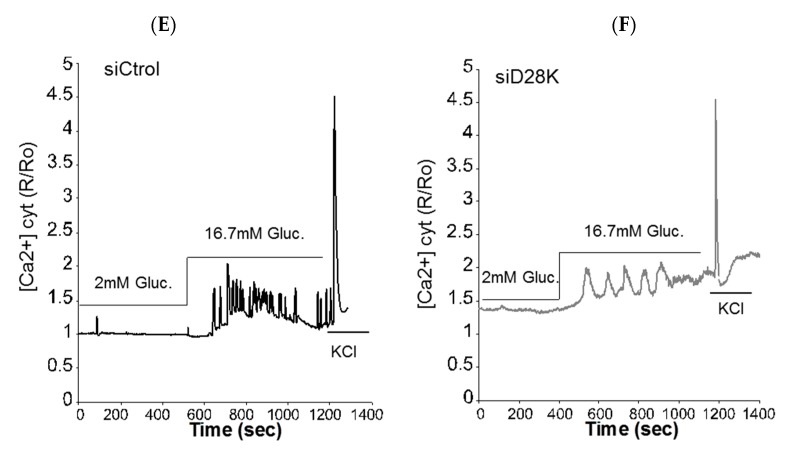
Isx9-rescued β cell function from serum withdrawal stress. (**A**) D28K subcellular distribution by immunoblotting in INS1 E cells cultured in vehicle or 10 µM Isx9 for 72 h, after starvation in Krebs Ringer HEPES (KRBH) (2 mM glucose) for 2 h (Basal) followed by stimulation with 16.7 mM glucose + 0.1 μM Ex-4 (Stim) for 10 min and pERK1/2 is used as a marker of β cell stimulation, TFII-I and ABCC9 are used as markers of nuclear and membrane fractions, respectively. (**B**) Insulin secretion in INS1 E cells after siD28K and Isx9 treatment for 48 h in basal (2 mM Gluc) and after 1 h stimulation (16.7 mM Gluc + 0.1 μM Ex-4) or (**C**) after culture for 48 h in control medium (10% FBS) or in SFM (no FBS) and in the presence or absence of 10 μM Isx9, * *p* < 0.05, ** *p* < 0.01. (**D**) Calcium traces (Fura-2) after glucose stimulation (Gluc.) in INS1 E cells after D28K Knockdown (siD28K) with and without Isx9. Represented as the mean of the 340/380 ratio values of an experiment done in triplicates. (**E**) A representative single cell calcium trace in INS1 E stably expressing the cytoplasmic Ca^2+^ biosensor CY 3.6 transfected with siCtrol or (**F**) siD28K after glucose stimulation and KCl depolarization.

D28K affects calcium influx through direct interaction with the L-type calcium channel subunit Ca_v_1.2 in vitro [14]. Subcellular fractionation of INS1 E cells after glucose stimulation, demonstrated by ERK1/2 phosphorylation, showed an increased plasma membrane enrichment of D28K in control and Isx9-treated cells by immunoblotting (Figure 5A) and confirmed by immunohistochemistry (Figure S6). D28K depletion increased insulin secretion, as previously shown [13], which is further increased by Isx9 treatment (Figure 5B). Reduced growth factor signaling in β cells impairs insulin secretion; Isx9 rescued β cells from dysfunction induced by chronic SFM treatment by significantly increasing GSIS (Figure 5C). D28K buffers [Ca^2+^]_i_ to reduce insulin secretion, however, D28K upregulation by Isx9 did not affect glucose-induced calcium mobilization (Figure S7A). This might result from a concomitant increase in calcium channel subunits (C_av_2.3, C_av_3.2) expression observed in Isx9-treated islets (Figure S1A). D28K knockdown increased glucose-induced calcium mobilization (Figure S7B), which is increased in combination with Isx9 treatment (siD28K + Isx9) (Figure 5D). In a single cell Ca^2+^ measurement, glucose induced an increase in the amplitude and frequency of cytosolic Ca^2+^ transients (Figure 5E). Knockdown of D28K reduced the frequency of Ca^2+^ transients and yielded broader peaks after glucose stimulation (Figure 5F). These data suggested that D28K contributes to the oscillatory frequency of glucose-induced Ca^2+^ transients.

### 2.5. Isx9 Effect on Human Islet Survival and Function after Transplantation in a Diabetic Mouse Model

**Figure 6:**
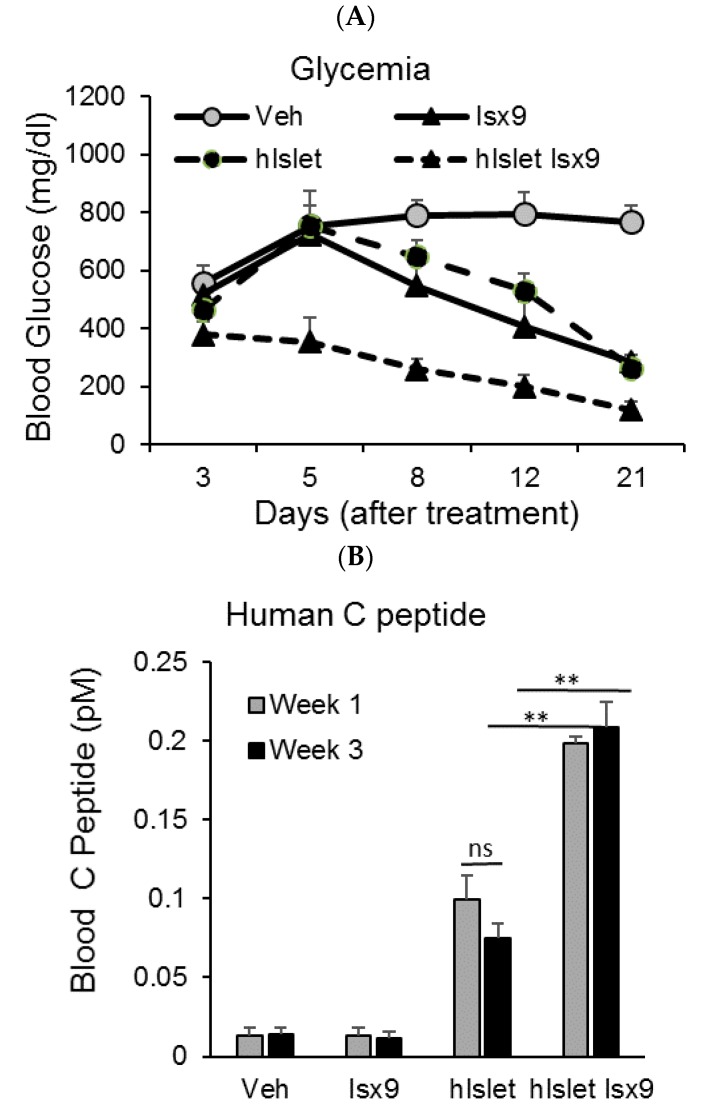
Isx9-improved human β cell function after transplantation into streptozotocin (STZ)-induced diabetic mice. (**A**) Time course of fed glycemia in STZ-induced diabetic mice after suboptimal human islet (500 IEQ) transplantation and/or Isx9 IP injection compared to vehicle sham-treated mice. Data represented as Mean ± SD, *n* = 4 per group. (**B**) Human C-peptide measured by ELISA (Mercodia, Uppsala, Sweden) in mouse plasma after human islet transplantation (hIslet) and/or Isx9 injection at week 1 (day 8) and week 3 (day 21), mean ± SD, ** *p* < 0.01.

In order to determine whether Isx9 can improve β cell function and glycemia in vivo, we used a streptozotocin (STZ)-induced diabetic mouse model characterized by an ablation of the murine islet β cells; these mice become insulin-dependent and develop diabetes. The mice were then transplanted with a suboptimal amount of human islets (hIslet) followed by daily injection with Isx9 or vehicle for up to three weeks. The control vehicle-treated sham transplanted mice after STZ treatment showed persistent hyperglycemia throughout the length of the study. However, the Isx9-injected sham transplanted mice showed glycemic improvement at the second week of injection (Figure 6A). The improvement of glycemia seen after transplantation with suboptimal numbers (500 IEQ) of human islets is significantly ameliorated with daily injection of Isx9 (hIslet + Isx9) over the course of three weeks (Figure 6A). Isx9 was previously reported to orchestrate the expression of factors regulating β cell function and differentiation in long-term cultured human islets [28]. To determine the effect of Isx9 on the transplanted islet function, we measured human C-peptide levels after the first and third week post-transplant by ELISA. Compared with vehicle treatment, Isx9 significantly increased human C-peptide level in transplanted mice fed ad libitum (Figure 6B). These data show that Isx9 promoted β cell maintenance and function in mice after transplantation of human islets.

## 3. Discussion

In the present study, we used Isx9, a small molecule regulator of β cell differentiation and function [28], to pharmacologically upregulate D28K expression in β cells and primary islets under conditions of pro-apoptotic cellular stress. D28K is known to have cytoprotective properties in both neurons and β cells in studies using transgenic overexpression mouse and genetic models [13,15,17]. Isx9 activation of D28K contributed to β cell survival under stress conditions by reducing caspase 3 activation. Isx9 induced calcium signaling, leading to activation of CN/NFAT in β cells. Among the NFAT target genes induced by Isx9, D28K is activated as early as 4 h following recruitment of p300, Creb, and NFATc1/NFATc2 to its promoter. Finally, Isx9 promoted human islet function after transplantation into a type 1 diabetic mouse model, opening new opportunities for the Isx9 molecule in diabetes care.

Isx9 increased [Ca^2+^]_i_ through activation of phospholipase β (PLCβ) and the L-type Ca^2+^ channels via an unidentified effector. Plasma membrane receptor activation of phospholipases raises [Ca^2+^]_i_ levels through the IP3 receptor activation in the endoplasmic reticulum and promotes a spike of [Ca^2+^]_i_ increase. Rise of [Ca^2+^]_i_ results in activation of many calmodulin (CaM)-dependent enzymes, including the phosphatase calcineurin, which dephosphorylates multiple phosphoserines on NFAT, leading to its nuclear translocation and activation [6]. We showed here that Isx9 increased D28K downstream of CN/NFAT. Disruption of the calcineurin subunit (Cnb1) in β cells induced an age-dependent diabetes characterized by reduced β cell proliferation and mass. This phenotype was rescued by overexpression of active NFATc1, indicating the importance of the calcineurin/NFAT in the regulation of β-cell survival and proliferation [8,41]. It has been previously shown in human islets that inhibition of CN/NFAT by FK505 reduced the expression of factors essential for insulin dense core granule formation and secretion and neonatal β cell proliferation [41], β cell mass, and function [42]. Our data show that Isx9 antagonized the inhibitory action of FK506 in an NFAT-reporter activity assay. This might result from the increase in NFATc1/c2/c4 expression and/or a direct effect of Isx9 on the CN phosphatase activity. However, the role of Isx9 on FK506 interaction with CN requires further investigation.

Activation of the CN/NFAT signaling pathway increased D28K expression and promoted β cell survival in response to serum withdrawal and inflammation. Serum deprivation (SFM) has been used as an in vitro model of ischemia and growth factor deprivation observed after organ transplantation [39]. Growth factor signaling, in particular through the insulin receptor or IGF1 receptor, promotes β cell function and replication [23,24,37]. Serum withdrawal with SFM induced intracellular reactive oxygen species, and increased sensitivity to cytotoxic stress and mitochondrial dysfunction in β cells [38,39,43]. SFM alters intracellular Ca^2+^ homeostasis through the release of Ca^2+^ ions from endoplasmic reticulum and activation of apoptotic factors such as caspases in hippocampal neurons and fibroblasts [43,44]. Through its buffering capacity and tight regulation of calcium influx, D28K inhibits the apoptotic signals triggered by excessive calcium signaling [45]. Several studies have previously demonstrated the cytoprotective properties of D28K, where overexpression of D28K in osteoblasts prevented apoptosis induced by TNF-α treatment [19] and inhibited caspase 3 activity in response to cytokine or glucocorticoid induced apoptosis in osteocytes [20] and pancreatic islet β-cells [15] with functional relevance in vivo [46]. In β cells, we observed plasma membrane enrichment D28K upon nutrient stimulation probably to control calcium channel activity. This observation goes in line with a previous report showing D28K interaction with the Ca_v_1.2 subunit of the voltage gated L-type calcium channel, leading to an increase of its voltage dependent inactivation in neurons [47] and possibly in β-cells [14]. It is likely that in β-cells, D28k plays an important role in the glucose induced pulsatile activation of the L-type calcium channel, a key step for the tight glucose regulated insulin secretion. Loss of D28K by siRNA led to broader and less frequent pulses of glucose induced Ca2^+^ entry, albeit the overall increase of cytosolic Ca^2+^ remained higher compared with control cells. It is likely that under physiological conditions, D28K acts as a gatekeeper through its possible interaction with the L-type calcium channel [14] to regulate glucose induced insulin secretion.

This current work shows the cytoprotective mechanism of Isx9 in β-cells involved in induction of D28K expression through increased recruitment of a transcriptional complex composed of NFAT, Creb, and p300 together with histone acetylation of the D28K promoter. Creb and NFAT regulate genes involved in β-cell function and survival [48]. Pancreatic β cells lacking Cnb1 failed to express NFAT targets required for replication, insulin storage, and secretion. Creb regulates cellular gene expression by binding to conserved Creb response elements (CRE) that occurs either as a palindrome (TGACGTCA) or a half site (CGTCA/TGACG) [49]. The D28K promoter contains one palindromic CRE site in the distal promoter and several half sites adjacent to NFAT binding site in the proximal promoter. We previously showed that Isx9 increased neuroendocrine gene expression in part through increased p300/CBP HAT activity, a major regulation of histone acetylation [28,29]. Similar to HDAC inhibitors like NaB [50], HAT activation increased core histone acetylation and activation of target genes and regulates β cell function and improved glucose homeostasis. Isx9 increased p300/CBP HAT activity, promoted the recruitment of p300 to the D28K promoter and histone H3 acetylation, leading to increased accessibility of the D28K promoter to transcription factors [51,52,53]. In the β cells, Isx9 facilitated the recruitment of the transcriptional complex composed of NFATc1/NFATc2, p300, and Creb1 to the D28K promoter. Isx9 increased Creb1 phosphorylation by Akt/PKB on Ser133, which in previous studies, was shown to increase its transcriptional activity and recruitment of co-activators p300/CBP to drive expression of the pro-survival genes such as bcl-2 and mcl-1 [54].

Our study sheds new light on signaling pathways regulated by Isx9 to abrogate apoptotic signals in β cell. Aside from increasing D28K expression, Isx9 also induced the expression of cytoprotective molecules such as CXCL12 and CXR4, which have the ability to stimulate regeneration and survival of β-cells in type 1 diabetes [55]. Furthermore, Isx9 upregulated the expression of several NFAT target genes involved in β cell replication and function such as Ccne1, Ccna2, Ccnd1, and the regulatory cyclin dependent kinase Cdk2. Therefore, some of the beneficial properties of Isx9 in β cell regeneration, as previously seen in a *PANIC*-ATTAC model [28] and in STZ treated mice shown here, might be mediated through the concomitant upregulation of D28K and cell cycle regulated genes. The limited supply of islet donors and the need for chronic treatment of recipients with immunosuppressors restrict the applicability and long-term efficacy of islet transplantation in patients with type 1 diabetes or type 2 diabetes who require insulin [56]. FK505 remains a highly effective immunosuppressant; however, it inhibits insulin secretion from human islet and is highly diabetogenic [57]. In β cells, Isx9 countered the inhibitory effect of FK506 in the CN/NFAT signaling pathway and might be used as a pharmacological tool to minimize the detrimental effect of calcineurin inhibitors in β cell function and survival. Further studies are needed to address the effect of Isx9 in CN/NFAT signaling, which is essential for the innate immune response in T-cells [58].

In conclusion, our results provide evidence that Isx9 can be used as a pharmacological tool to protect against stress-induced β cell death and dysfunction in part through transcriptional upregulation of D28K. Here, we confirmed the previously described anti-apoptotic properties of D28K from genetic models. Moreover, we propose a novel mechanism by which Isx9 regulates D28K expression in pancreatic β cells through activation of the CN/NFAT signaling together with Creb1 and p300 recruitment to the D28K promoter. Isx9 as a CN/NFAT activator promoted β cell function and improved human islet function (Insulin secretion) after transplantation in an STZ-induced diabetic mouse model, as well as mouse islet function after STZ treatment. While not directly tested in the islet transplantation study, the beneficial effects of D28K on β-cell survival in response to stress could promote transplanted islet replication and maintenance. Isx9 offers new therapeutic potentials in diabetes that need to be further characterized.

## 4. Materials and Methods

### 4.1. Cell Culture and Preparation of Mouse Islets

Rodent β cell lines MIN6 and INS1 E were maintained in DMEM and RPMI-1640 medium (Life Technologies, Carlsbad, CA, USA), respectively [59]. The complete medium supplemented with 10% FBS was used as a control culture condition compared with serum-free medium (SFM) culture condition without FBS. The calcineurin inhibitor FK506 and the small molecule *N*-cyclopropyl-5-(thiophen-2-yl)-isoxazole-3-carboximide (Isx9, CAS No. 832115-62-5, Tocris, Bristol, UK) were used as indicated at a final concentration of 0.3 µM and 10 µM, respectively, and 0.01% DMSO is used as vehicle control. Cells were treated with cytokines mix (50 U/mL IL-1β, 1000 U/mL TNFα, and 1000 U/mL IFNγ) in culture media for up to 48 h. All cultures were kept in a humidified atmosphere at 37 °C and 5% CO_2_. Mouse islets were isolated by hand-picking after collagenase 4 digestion (422 U/mL, Worthington, NJ, USA) of pancreas, as previously described [60], and were maintained overnight in RPMI-1640 complete medium [28]. Islets were cultured in monolayer in plates coated with extracellular matrix (ECM) derived from bovine corneal endothelial cells (Novamed, Jerusalem, Israel).

### 4.2. Immunofluorescence and Confocal Microscopy

Islets isolated from wild type mice were cultured on glass coverslips coated with a laminin-rich extracellular matrix (ECM) produced from rat bladder 804 G carcinoma cell line. After treatment with 10 μM Isx9 for 48 h or vehicle (DMSO), islets were fixed in 4% PFA in PBS for 30 min and permeabilized in 0.15% Triton X-100 and blocked for 1 h in PBS containing 10% normal donkey serum with 0.05% triton X-100. Islets were then incubated with primary antibodies at 4 °C overnight, washed with PBS containing 0.2% Tween-20, and then incubated with corresponding donkey secondary antibodies diluted in 10% normal donkey serum in PBS Tween-20 for 2 h at room temperature. Microscope slides were individually imaged using an SP8 confocal microscope (Leica, Wetzlar, Germany).

### 4.3. Western Blotting and Antibodies

Protein samples from whole cell extracts or subcellular fractionation (ThermoFisher Scientific, Waltham, MA, USA) were isolated accordingly. Samples were loaded in SDS-PAGE after BCA protein quantification (Bio-Rad, Hercules, CA, USA). The protein was transferred from the gel onto nitrocellulose membranes (Bio-Rad) and incubated overnight at 4 °C with the following primary antibodies: anti-Calbindin D28K (Santa Cruz Biotechnology, Dallas, TX, USA, sc-7691, sc-365360; 1:400), anti-TFII-1 (Santa Cruz Biotechnology sc-28716, 1:500), and NFATc1 (Pierce MA3-024, Santa Cruz Biotechnology, 1:400; sc-7294). Fluorescent-labeled secondary antibodies (Li-Cor, Lincoln, NE, USA) were incubated for 1 h at room temperature followed by washes on an orbital shaker with TBS 0.05% Tween-20. Blots were then scanned with a Li-Cor Odyssey scanner (Li-Cor, Lincoln, NE, USA).

### 4.4. Luciferase Assays

NFAT transcriptional activity was measured using the PathDetect NFAT cis-Reporting System (Agilent Technologies, La Jolla, CA, USA) containing four xNFAT binding sites (GGA**GGAAAAACTGTTTCA**TACAGAAGGCGT) upstream of the firefly luciferase reporter gene, co-transfected with the pRL-SV40-Renilla luciferase as a normalizer. All transient transfection experiments were done with cells 70% to 80% confluent in 96-well plates using Lipofectamine LTX with Plus reagent (Life Technologies), following the manufacturer’s recommendation. Luciferase activity was measured using the dual luciferase kit (Promega, Madison, WI, USA) 24 h after treatment with Isx9 +/− FK506 using the Cytation 3 plate reader (Biotek, Winooski, VT, USA).

### 4.5. Chromatin Immunoprecipitation (ChIP) Assays

INS1 E cells grown to 80% to 90% confluency were treated with 10 µM Isx9 for up to 24 h. ChIP assays were performed as previously reported [10,59]. Briefly, cells were cross-linked with 4% paraformaldehyde (PFA) and the chromatin was fragmented by sonication using the Covaris E220 ultrasonicator (Woburn, MA, USA, using TruChip protocol) in an ice-cold water bath. Antibodies against NFATc1 (MA3-024), Creb (PA1-850), and p300 (MA1-16608) purchased from Thermofisher Scientific were immobilized on protein A-Sepharose beads and used to precipitate protein/DNA complexes. The bound DNA fragments were quantified by real-time qPCR using SYBR Green Master Mix (Applied BioSystems, Foster City, CA, USA) with primer sets to amplify proximal and distal promoter [10]. Recruitment of transcription factors to chromatin was expressed relative to input. Primer sequences are listed in Supplemental Table S1.

### 4.6. RNA Isolation and qPCR

Total RNA was prepared by miniRNA kit Plus (QIAGEN, Hilden, Germany). RT-PCR was performed with the Applied High Capacity cDNA Synthesis kit (ThermoFisher Scientific) and cDNA was used for qPCR analysis. The target gene expression was evaluated using Power SYBR Green PCR Master Mix (Applied Biosystems, Foster City, CA, USA). PCR was carried on LightCycler 480 Real-Time PCR Systems (Roche, Basel, Switzerland) using a LightCycler 1536 SYBR green (Roche) [61]. Transcript levels were normalized to cyclophilin B. Relative fold change in expression was calculated using the ΔC_T_ method. For relative transcript quantification, each cDNA sample was run on a four-point standard curve to assure a PCR efficiency of ≥95%. Primer sequences are listed in Table S2.

### 4.7. Calbindin D28K siRNA Knockdown

Endogenous D28K expression was knocked down in INS1 E by siRNA reverse transfection using rodent Silencer Select siRNA oligos targeting rodent Calbindin D28K gene (siRNA ID: s136343, sense 5′-GGAAUUGGAUAUUAACAAUtt-3′ antisense 5′-AUUGUUAAUAUCCAAUUCCtg-3′ Ambion, ThermoFisher Scientific) using lipofectamine RNAiMax (ThermoFisher Scientific, Waltham, MA, USA) in OptiMEM medium (Life Technologies) according to manufacturer’s instructions.

### 4.8. Insulin Secretion Assay

INS1 E cells treated according to experimental conditions were starved for 1 h in Krebs Ringer HEPES (KRBH) buffer in low glucose (2.8 mM) followed by incubation in 2.8 mM glucose for 1 h (Basal) before stimulating with 16.7 mM glucose for 1 h. Insulin and content were determined by ELISA (ALPCO Diagnostics, Salem, NH, USA) in nanogram per milliliter (ng/mL) and normalized with total protein.

### 4.9. Calcineurin Activity

Calcineurin activity was measured from MIN6 cell extracts after 2 h in KRBH 2.8 mM glucose and after treatment with FK506 or increasing concentrations of Isx9 using the colorimetric calcineurin activity assay kit (Enzo Life Sciences, Farmingdale, NY, USA). Calcineurin activity was determined as the difference between total phosphatase activities minus the phosphatase activity in the presence of 10 mM EGTA that blocks endogenous calcineurin activity. Data were determined as percentage of control after normalization of absorbance at 620 nm using a plate reader (Cytation3 Biotek, Winooski, VT, USA).

### 4.10. Calcium Measurement

Intracellular calcium [Ca^2+^]_i_ influx was measured in INS1 E cell populations as described previously [62]. Briefly, cells plated in black-walled 96-well plates coated with the 804 G extracellular matrix and loaded with 5 μM Fura-2 AM (Life Technologies). Cells were stimulated with 16.7 mM glucose in KRBH, and changes in intracellular calcium levels were assessed every 0.74 s for 2 min by dual excitation of Fura-2 at 340/11 and 380/20 nm (center/band pass) and emission at 508/20 nm using the Cytation 3 multi-mode micro plate reader (Biotek, Winooski, VT, USA). Measurements were performed in triplicate for each individual experiment at least three times. Single cell measurement of cytosolic Ca^2+^ was done using INS1 E cells stably expressing the genetically encoded cameleon Ca^2+^ sensors YC3.6 cyto (INS1 E-YC3.6), as described previously [63,64]. Cells were imaged on a DMI6000 B inverted fluorescence microscope using an HCX PL APO 63 x/1.40–0.60 NA oil immersion objective (Leica Microsystems, Wetzlar, Germany) with an Evolve 512 back-illuminated CCD with 16 × 16 pixels camera (Photometrics, Tucson, AZ, USA) and excited at 430 nm through a BP436/20 filter. The two emission images were acquired with BP480/40 and BP535/30 emission filters. Fluorescence ratios were calculated in MetaFluor 7.0 (Meta Imaging Series) and analyzed in Excel (Microsoft, Redmond, WA, USA) and GraphPad Prism 5 (GraphPad). Images were taken every 2 s.

### 4.11. Cell Viability Assay

INS1 E viability after treatment was assessed using Alamar blue (ThermoFisher Scientific, Waltham, CA, USA) according to manufacturer’s recommendations [65]. Control cells treated with vehicle were normalized to 100% and cell survival was calculated as a relative percentage of the control.

### 4.12. Mouse Studies

Animals (*n* = 4 per group) were housed on a 12 h light/dark cycle at the Animal Resources Center at UT Southwestern Medical Center (IACUC animal protocol number 2010–0273) under humidity- and temperature-controlled conditions. NOD-SCID mice (Charles River, Wilmington, MA, USA) fed standard chow diet with free access to water were rendered diabetic after streptozotocin (STZ, 75 mg/Kg) IP injection-induced destruction of β cells. Suboptimal numbers of human islets (500 IEQ) were transplanted into the kidney capsule of the diabetic mice and Isx9 or vehicle were administered as previously described [29]. Briefly, mice were injected intraperitoneally once daily with 16 mg/kg Isx9 (dissolved in 20% hydroxypropyl-β-cyclodextrin (hpcd) (AC29756-5000) (ThermoFisher Scientific, Waltham, MA USA) at 2 mg/mL final concentration) daily or equivalent amount of hpcd (vehicle). Blood glucose was monitored up to three weeks after STZ injection. Circulating human C-peptide levels in treated mice were measured by ELISA (Mercodia, Uppsala, Sweden).

### 4.13. Statistics

All experiments were performed at least twice in triplicates for each condition. Data are reported as the mean ± SEM. Data were analyzed by the Student’s *t*-test for paired observations. When comparing three or more means, analysis of variance (ANOVA) was applied followed by Dunnett’s multiple comparison tests. All analyses were performed using the Prism 6.0 program (Graph Pad Software, San Diego, CA, USA). A value of *p* < 0.05 was considered significant.

## Supplementary Materials

The supplementary materials are available online at http://www.mdpi.com/1422-0067/19/9/2542/s1.
