# Supplementary material for: Isx9 Regulates Calbindin D28K Expression in Pancreatic β Cells and Promotes β Cell Survival and Function
Source: Int J Mol Sci. 2018 Aug 27;19(9):2542. doi: 10.3390/ijms19092542 (PMC6165483; doi:10.3390/ijms19092542)
Supplement: Supplementary file 1 [file ijms-19-02542-s001.zip › ijms-340562 - supplementary material/ijms-340562-supp-fig.pdf]

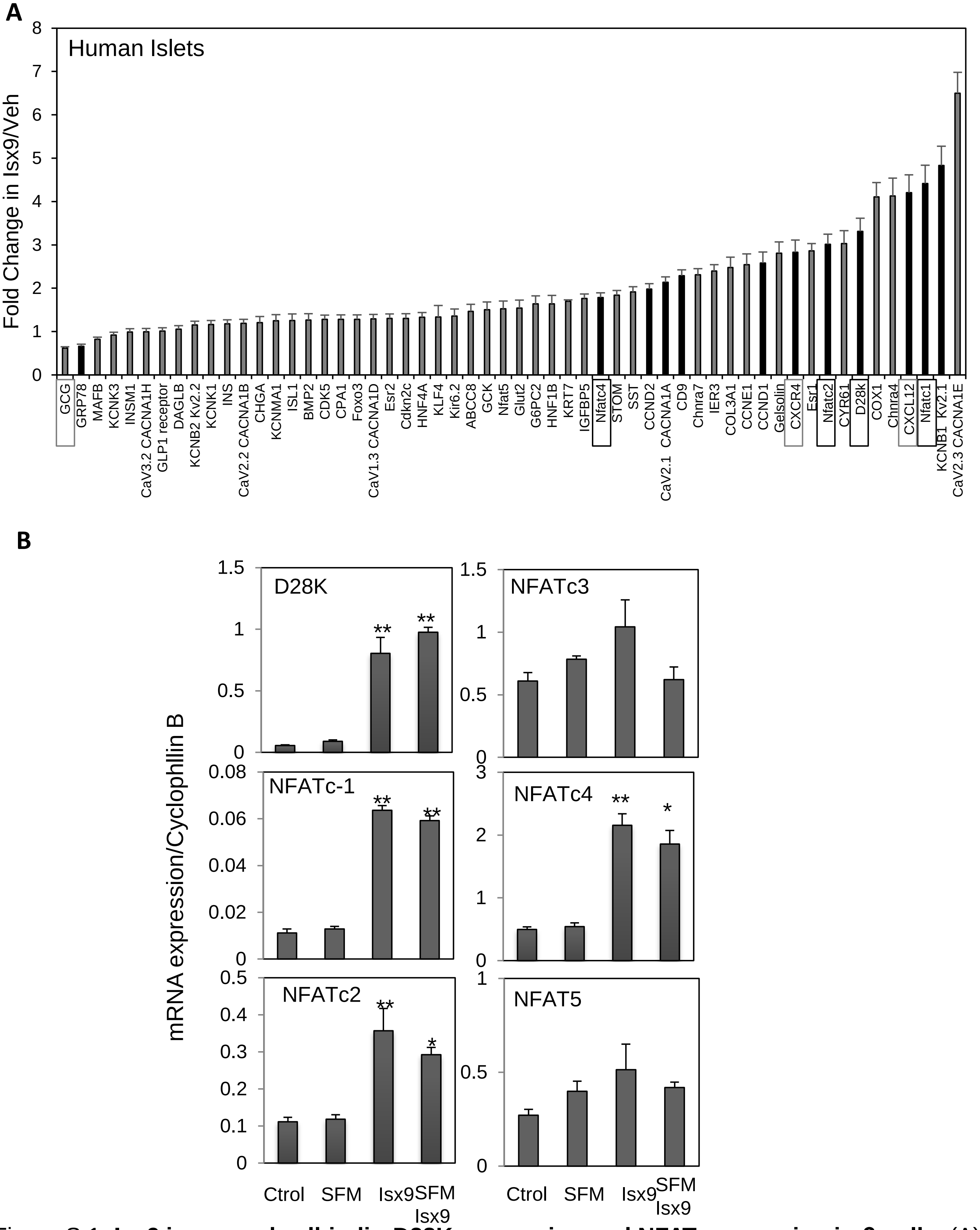

Figure S 1. **Isx9 increased calbindin-D28K expression and NFAT expression in β-cells**. (A) Expression of selected genes induced by 10 μ Isx9 for 24 hrs in human islets using the NanoString nCounter gene expression array (B) Expression by qPCR of NFATc1,c2,c3,c4 and NFAT5 in INS1E cells after treatment with control 10% FBS(Ctrl) or SFM in the presence or absence of 10μM Isx9 for 24hrs. *p<0.05 and **p<0.01 compared to control (Ctrl).

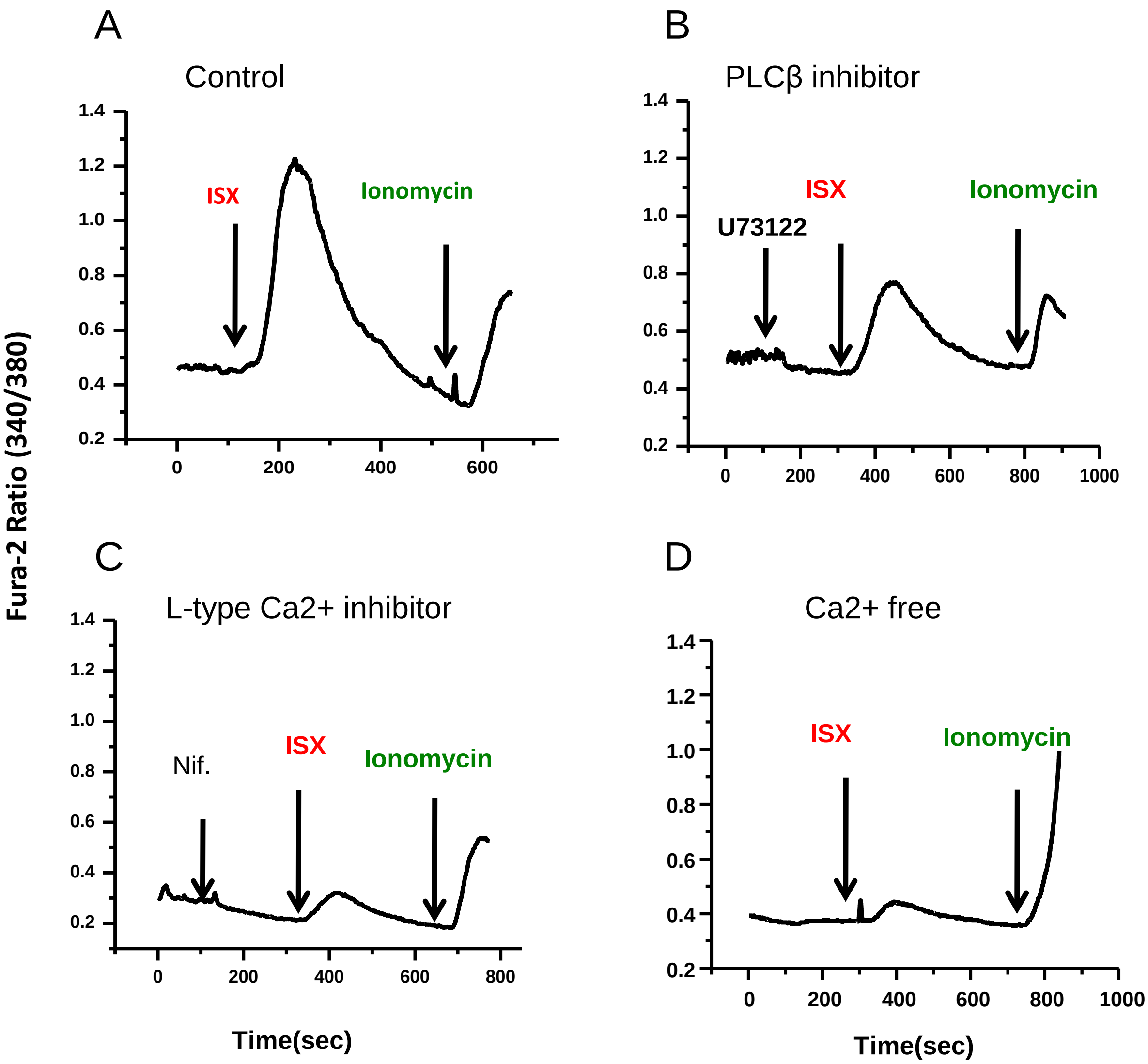

Figure S2. Isx9 acutely induced calcium entry through L-type Calcium channel in MIN6 cells. (A) In control media, Isx9 increased Ca2+mobilization, the calcium ionophore Ionomycin is used as a positive control. (B) Inhibition of the PLCβ downstream of Gαq downstream of GPCR reduced calcium entry induced by Isx9 in half. (C) Nifedipine (Nif.) inhibition of L-type calcium channel reduced significantly Isx9 induced Ca2+ entry to levels similar to induction in Calcium free medium (D) representing release from internal stores. Representatives calcium traces from singles cells, at least 20 cells were measured per condition and experiment is performed at least twice.

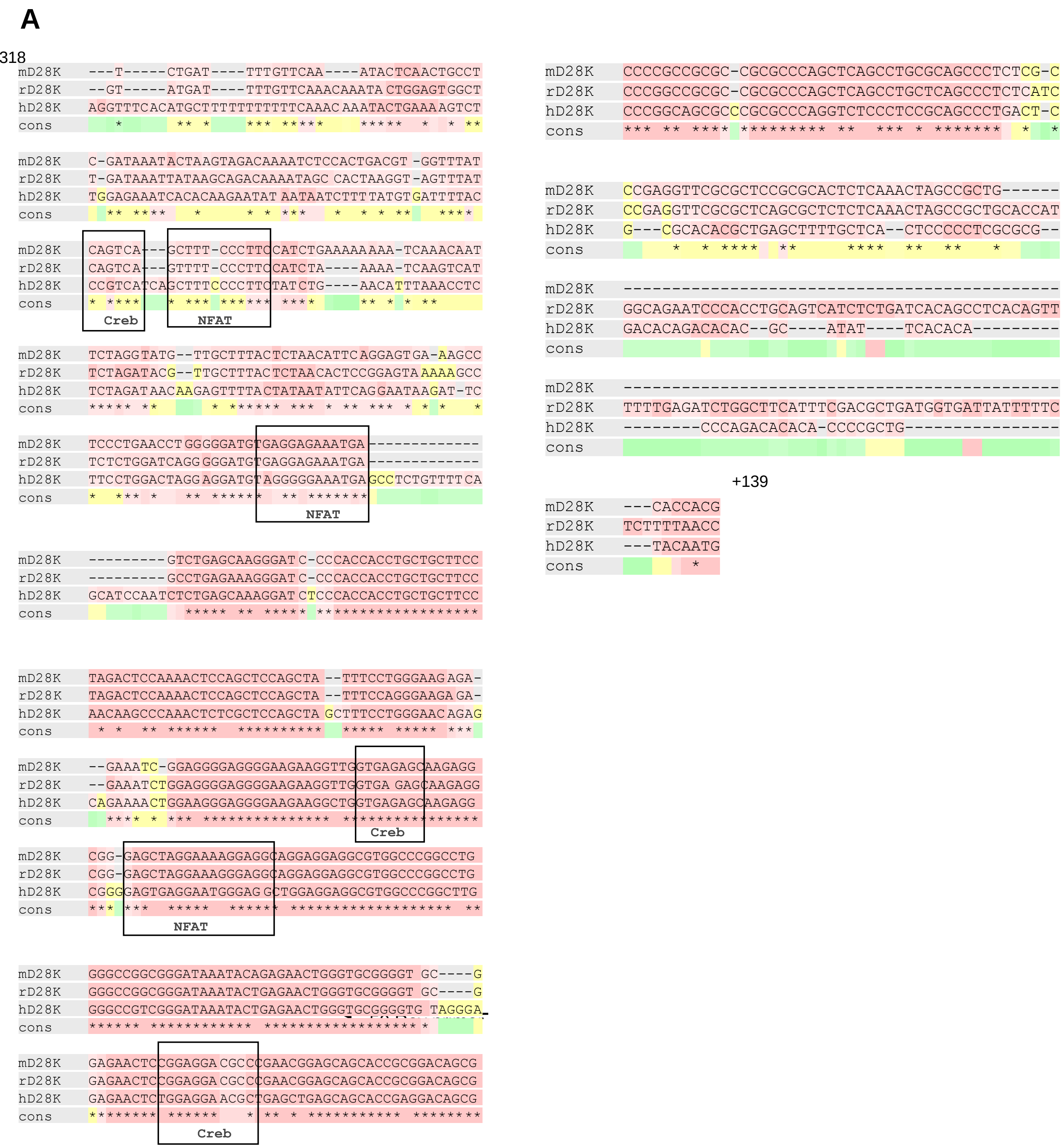

Figure S3. Multiple sequence alignment and conserved sequences of the mouse, rat and human D28K proximal promoter using the Tcoffee server (http://tcoffee.vital-it.ch/apps/tcoffee/result?rid=50413899). NFAT and Creb binding sites are highlight in the boxes.

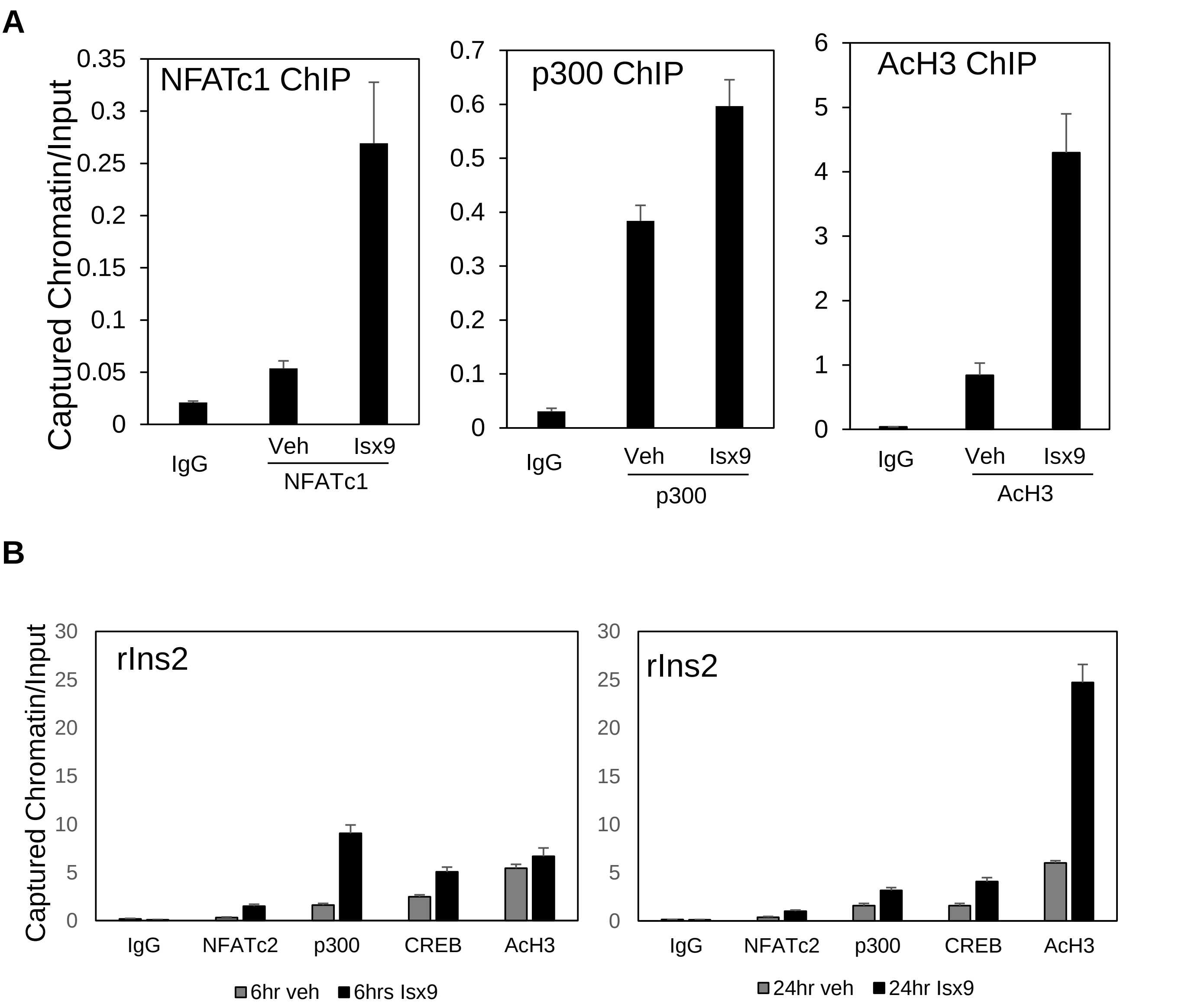

Figure S4. Isx9 increased recruitment of NFATc1 Creb1 and p300and to the mouse D28K and rat insulin 2 (rIns2) promoter by ChIP-assay. ((**A**) Recruitment of NFATc1, p300 and acetylated histone H3 (AcH3 K9/14) of the proximal mouse D28K promoter (-36/+139) by ChIP-assay after treatment of MIN6 cells with Isx9 for 24hrs. (**B**) Chromatin enrichment of NFATc2, Creb1, p300 and acetylated histone H3 (AcH3 K9/14) to the rat insulin promoter in INS1E cells treated with 10 µM Isx9 for 6hrs or for 24hrs (-239/-92).

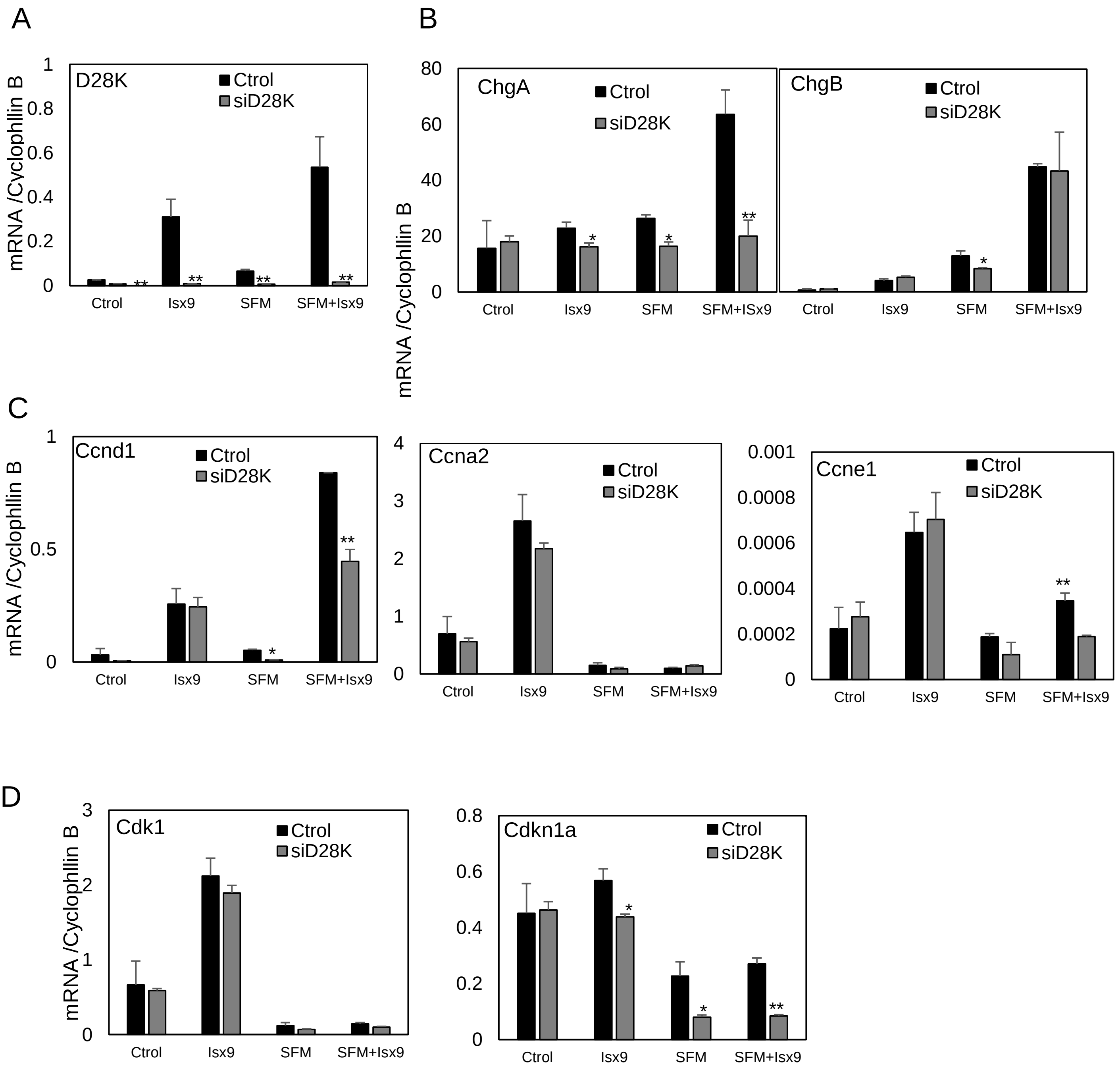

Figure S5. Effect of D28K and Isx9 in functional gene expression after response to serum withdrawal (SFM) in INS1E cells. (A) Expression of D28K and selected genes involved in β cell function (B) Chromogranin A, Chromogranin B and (C) cell cycle proteins like the cyclins and (D) cyclin dependent kinases in INS1E cells after D28K knockdown and treatment with Isx9 and Calbindin expression in INS1E cells treated with vehicle or 10uM Isx9 for 24 hrs. *p<0.05, **p<0.01 Data are represented as mean and standard deviation of n=3 cell after D28K siRNA knockdown and 10 μM Isx9 treatment for 48hrs. Data are represented of n=3 Mean ± SD *p<0.05, **p<0.01 control versus siD28K.

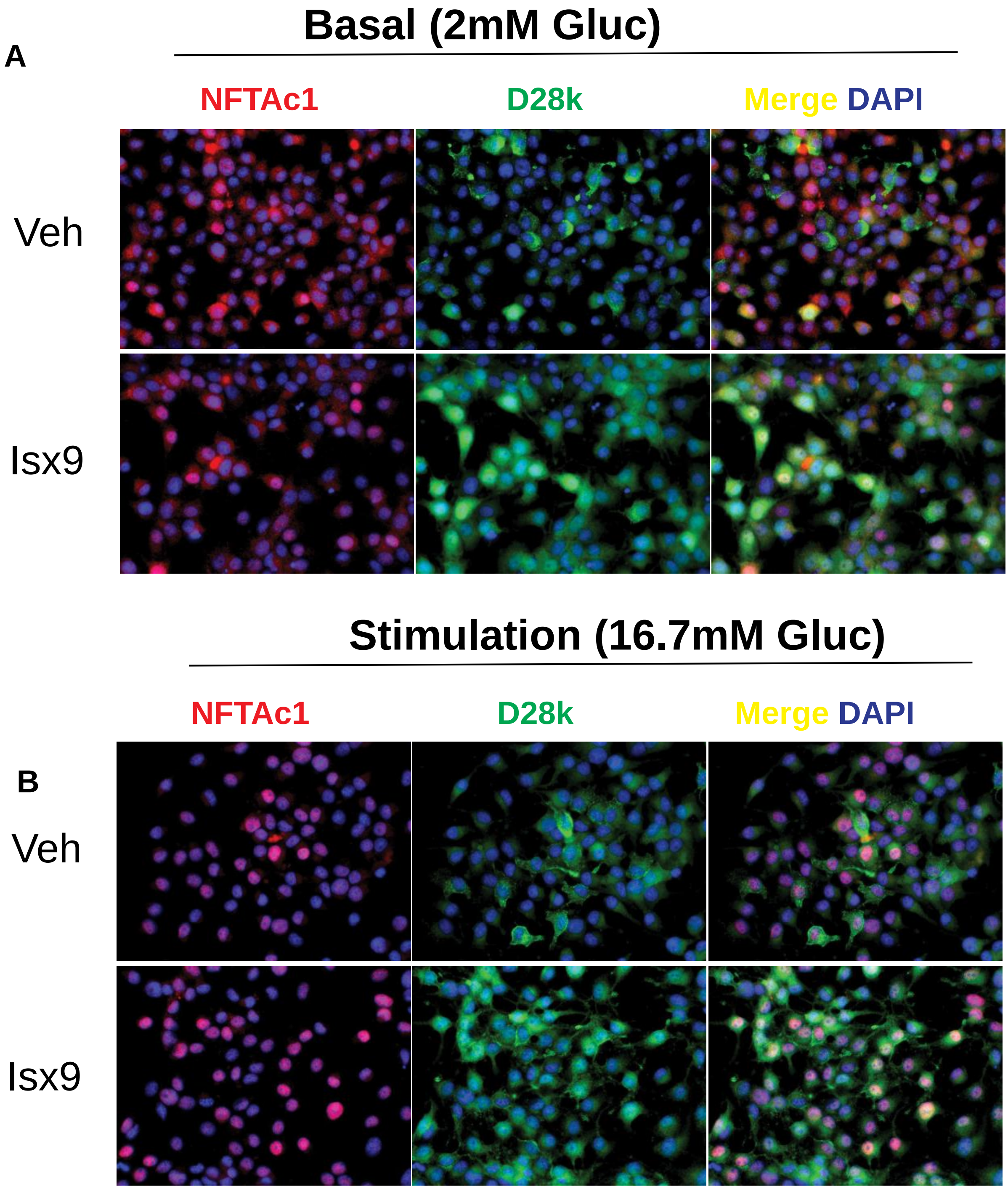

Figure S6. INS1E cells cultured on glass coverslip cells were treated with Vehicle (DMSO) or 10μM Isx9 for 72hrs. Cells were starved for 2hrs in KRBH 2mM Gluc (Basal) (A) followed by stimulation with 16.7mM Gluc. + 0.1μM Exendin-4 for 10 min ((B). After fixation (4% PFA), permeabilization (0.25% Triton X-100) and blocking (5% Donkey Serum; 1% BSA; 0.05% Triton) cell were incubated at 4°C with mouse anti-NFATc1 (Thermo, 1/50) and goat anti-Calbindin1 (D28K) overnight. Secondary antibodies (Alexa Fluor, Molecular Probes) and DAPI were diluted and incubated in blocking buffer 1 hour at RT. Coverslips were mounted in Pro Diamond Mounting Media (Thermo Scientific) on microscopy slide and images were acquired using the Leica SP8 confocal microscope.

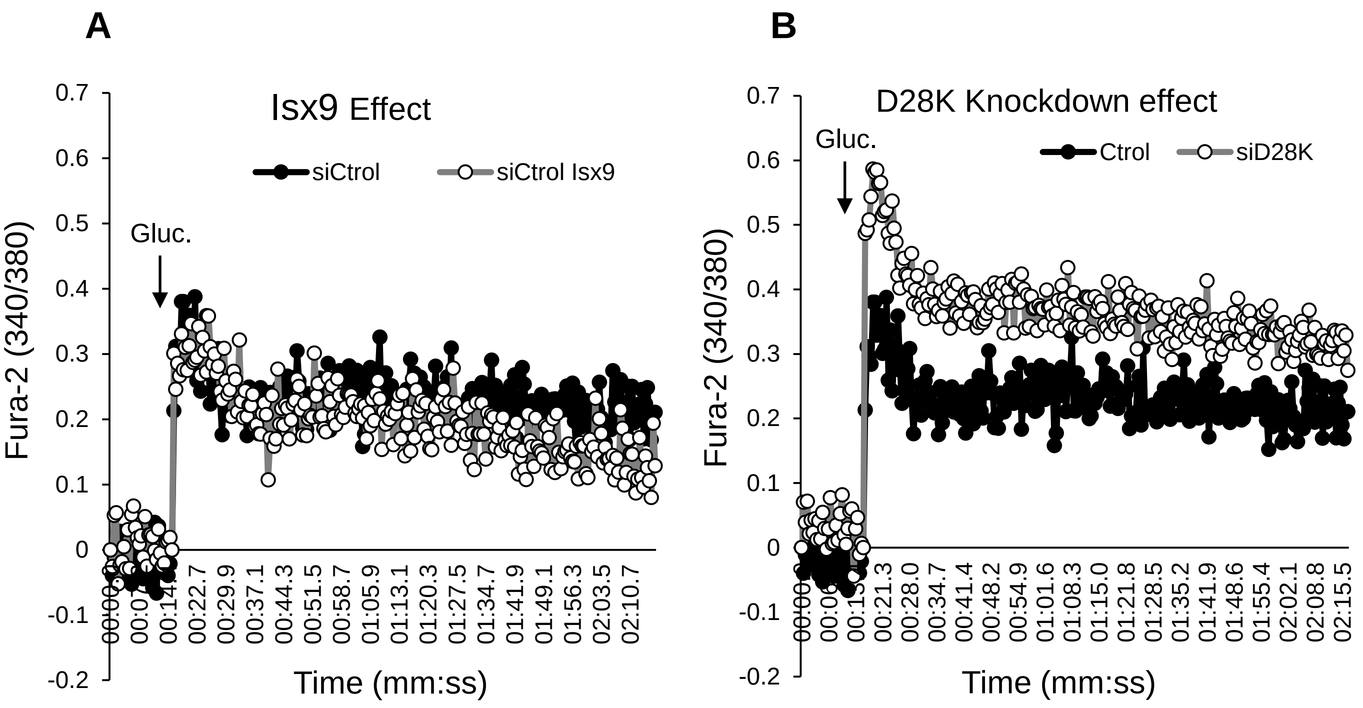

Figure S7. INS1E cells plated on black walled 96-well plates coated with 804G extracellular matrix coated. After siRNA knockdown (72hrs) and/or Isx9 (48 hrs) treatment, cells were washed with KRBH and loaded with 5 µM Fura-2AM (Life Technologies) and intracellular calcium influx was measured after stimulation with 16.7 mM glucose (Gluc.) . (A) Calcium traces from INS1E cells after Isx9 treatment transfected or (B) after siRNA knockdown of D28K (siD28). Represented as mean of the 340/380 ratio values of 3 independent experiments.
