# Supplementary material for: Isx9 Regulates Calbindin D28K Expression in Pancreatic β Cells and Promotes β Cell Survival and Function
Source: Int J Mol Sci. 2018 Aug 27;19(9):2542. doi: 10.3390/ijms19092542 (PMC6165483; doi:10.3390/ijms19092542)
Supplement: Supplementary file 1 [file ijms-19-02542-s001.zip › ijms-340562 - supplementary material/ijms-340562-supp-table.pdf]

Supplemental Table 1 : Primers used in ChIP-assay

**Primer name**	**Forward primer**	**Reverse primer**	**Fragment Amplified**
**rCalb-ChIP-p1**	5'-GAGCGGAGAAGAGATGGGTA-3'	5'-CTCCCTGCCTTGCTCTGAAT-3'	-6777/-6536
**rCalb-ChIP-p2**	5'-AATGAGCATGGTGACATGGA-3'	5'-TGCAGATGTTTCAGGGATGA-3'	-5435/-5310
**rCalb-ChIP-p3**	5'-TTCTCATTTTGGAGTCTTTTTCTC-3'	5'-TTTGGTCGGTTGCTTTACAT-3'	-2940/-2970
**rCalb-ChIP-p4**	5'-CCTGAGACTGAACGGAGAGG-3'	5'-CACCCTTTGGACTTGCTTGT-3'	-1396/-1240
**rCalb-ChIP-p5**	5'-CCACCTGCTGCTTCCTAGAC-3'	5'-CCGCACCCAGTTCTCAGTAT-3'	-3206/-93
**rINS**	5'-CTGGGAAATGAGGTGGAAAA -3'	5'-CGTGGTGCAGCGGCTAGTTTG-3'	-140/+139
**mCalbindin-ChIP-p7**	5'-CACCCGGGTACCGTGCGGAGAACTCCGGAGGAC-3'	5'-CGTGGTGCAGCGGCTAGTTTG-3'	-36/+139

Supplemental Table 2: Primers pairs used for qPCR gene expression in rat mouse and human samples.

Rat qPCR primers

Primer name	Forward Primer	Reverse Primer
D28K	AGGAAAGGAGCTGCAGAACT	CCCATCATCTCTCTGCCCAT
NFATc1	CCTAATTCCCTGGTGCTGGA	GCTTCCCGTTGCAGACATAG
NFATc2	ATCACCGAGCCCACTATGAG	GCTTGTTCTCCATGTAGCCG
NFATc3	GTGGCCATCCTGTTGTGAAG	GCACCTGGTAAAATGCGTGA
NFATc4	GAGGGGAGGAGCTAGTGTTG	TGGTCAGAGTCAGTGTCACC
NFAT5	TCCTACGGTGAAGCTGGAAG	TGCCTTCGATGTCCACTTCT
GRP78	CCGAGGCGTATTTGGGAAAG	ATAGTGCCAGCATCCTTGGT
ChgA	ACAACACAGCAGCTTTGAGG	GTCCTTCTGTGGTTCCCTCA
ChgB	GAAATGGTGACTCGCTGCAT	ACTCTTCCTCAGGACTTGCC
Ccnd1	GCGTACCCTGACACCAATCT	GGCTCCAGAGACAAGAAACG
Ccnd2	TTACCTGGACCGTTTCTTGG	TGCTCAATGAAGTCGTGAGG
Ccne1	GACAGCTAGCGCGGTGTAG	GTTGCTGTGGTCCTTCGAGT
Cdk1	CCGAAATCTGCCAGTTTGAT	CTGGCCAGTTCATGGATTCT
Cdk2	CATTCCTCTTCCCCTCATCA	GTACGGACAGGGACTCCAAA
Cdkn1a/p21	CTTGTCGCTGTCTTGCACTC	AGGCAGAAGATGGGGAAGAG

Mouse qPCR primers

Primer name	Forward Primer	Reverse Primer
D28K	TCTGGCTTCATTTCGACGCTG	ACAAAGGATTTCATTTCCGGTGA
NFATc1	CCGTTGCTTCCAGAAAATAACA	TGTGGGATGTGAACTCGGAA
NFATc2	CCAATCAGTCGGGCTCCTAT	GCTTGTTCTCCATGTAGCCG
NFATc3	GTGGCCATCCTGTTGTGAAG	AGTAATCCGATGCACCTGGT
NFATc4	ATCCCCGAGTACAGCAACAA	GATGAGTCTGGTAGGGGCTC
NFAT5	TGGGCGAAATACAACTCCCT	CATCGGCATTCCTCAGCTTC
GRP78	ACTTGGGGACCACCTATTCCT	ATCGCCAATCAGACGCTCC
BDNF	CACTTTTGAGCACGTGATCGA	CGTTGGGCCGAACCTTCT

Human qPCR primers

Primer name	Forward Primer	Reverse Primer
NFATc1	GGACATCGGGAGGAAGAACA	ACAGGACCATCTTCTTCCCG
D28k	TCATCCCTCATCACAGCCTC	AGTTCTGCAGCTCCTTTCCT
